# Are we ready? Emergency unit capacity at selected district level hospitals in Lusaka Province, Zambia: Barriers and facilitators for improving trauma care: a mixed methods approach

**DOI:** 10.1371/journal.pgph.0004382

**Published:** 2025-05-09

**Authors:** Penelope Kantu Machona, Joseph Mumba Zulu, Mpundu Makasa, Eivind Meland, Thomas Mildestvedt

**Affiliations:** 1 Department of Surgery, University of Zambia, School of Medicine, Lusaka, Zambia; 2 Department of Health Promotion, School of Public Health, University of Zambia, Lusaka, Zambia; 3 Department of Health Policy and Management, School of Public Health, University of Zambia, Lusaka, Zambia; 4 Department of Community and Family Medicine, School of Public Health, University of Zambia, Lusaka, Zambia; 5 Department of Global Public Health and Primary Care, University of Bergen, Bergen, Norway; Royal Infirmary of Edinburgh, UNITED KINGDOM OF GREAT BRITAIN AND NORTHERN IRELAND

## Abstract

The increasing burden of road traffic injuries (RTIs) has become a public health concern in Zambia for the last five years. Little is known about the capacity and determinants of emergency care at the point of first contact in a country without coordinated pre-hospital and emergency medical services (EMS). Evaluation of the in-hospital emergency trauma care for RTIs is critical. This study sought to assess the emergency care at the district level hospitals to effectively manage RTIs, using the WHO Hospital Emergency Assessment Tool (HEAT), and identify the barriers and facilitators. A mixed-methods approach was employed at ten facilities in Lusaka Province between May 2023 and September 2023. Quantitative data were collected using the WHO HEAT instrument on facility matrices, infrastructure and equipment, human resources, diagnostic and clinical services, and signal functions. Thirty-five interviews were conducted with emergency unit healthcare providers at these facilities to identify the barriers and facilitators to care. The WHO tool guided inductive and deductive thematic analysis. Emergency care services were available 24 hours a day, with a mean bed capacity of 4.7 for the ten (10) facilities sampled. Eight hospitals had a designated emergency unit and three had no triage area. Only four hospitals had a core emergency trauma team. The key barriers to care were shortage of equipment and consumables, a lack of skills and specialist services to perform signal functions, and inadequate ambulance services. However, supportive and committed leadership, team cohesiveness, interdepartmental collaboration, motivated staff, and skills transfer from seniors emerged as the facilitators to care. Lusaka Province is moderately prepared for the increasing number of emergency trauma cases. To strengthen emergency trauma care; capacity building for human resource in triage, resuscitation, and trauma interventions for the initial care is integral. Deliberate action through budgetary support for infrastructure development, emergency equipment procurement, increased ambulance service availability, and recruitment of skilled human resources is timely.

## Introduction

Injury is a leading cause of morbidity and mortality globally accounting for about six million deaths per year with 90% of these occurring in low- and middle-income countries (LMICs) [1,2]. The Global Burden of Disease (GBD) study, found a reduction in the rates of death- or disability-adjusted life years (DALYs), from Road Traffic Injuries (RTIs), mainly in high-income countries, whereas low- and medium-income countries (LMICs) showed an increase [3]. This increasing burden calls for the strengthening of healthcare systems in LMICs to improve access to high-quality emergency care for vulnerable populations [4,5]. Identifying gaps in the delivery of emergency care is essential for formulating interventions targeted at key areas in the existing model for an LMIC with competing budgetary needs [6,7]. However, the lack of healthcare system evaluation poses a challenge to priority setting and policy direction for optimal care of these patients [8]. Improved emergency trauma care systems have been shown to improve outcomes and reduce mortality by over two million lives if implemented to standards of care comparable to those of developed countries [9,10]. An accident victim may lose their life or develop secondary complications in the absence of prompt detection and diagnosis of their life-threatening conditions at admission to a health facility [11]. Primary care facilities are the first point of contact in LMICs; however, a lack of capacity leads to poor health outcomes in an overburdened system concerning both communicable and non-communicable diseases [12].

All United Nations (UN) member states have agreed to achieve universal health coverage (UHC) by 2030 by creating opportunities to address the Sustainable Development Goals (SDGs), which include supporting injury control initiatives and organized trauma care for the injured, reducing injury burden, and strengthening health systems [13]. To strengthen healthcare delivery, good leadership and coordination should be at the core of implementation at all levels, from national, regional, and district, down to individual healthcare facilities for every system [14].

This study is set in Zambia, a developing country located in the southern part of the sub-Saharan Africa region with a population currently of 19.6 million people; Lusaka Province has the largest population standing at 3.1 million out of all the ten provinces [15,16]. The estimated mortality rate from road traffic crashes for Lusaka Province in 2020 was 53 deaths per 100,000 population and 37.9 deaths per 100,000 population for the entire country [17]. Zambia recorded an increase in the number of RTIs by eight (8) percent in the year 2022 compared to 2023: slight injuries 10,212 from 9,234 and 5,828–6,027. Lusaka Province alone accounted for 55.1% of fatalities in the national total [18]. The health service delivery system is organized into four levels in a pyramidal structure, the base level being Primary Health Care (PHC) services at the district level (health posts, health centres, mini-hospitals, and level-one hospitals) followed by second level (provincial) and third-level hospitals, the latter being national referral units (tertiary), while the fourth level comprises national referral but specialized hospitals. Majority of facilitate despite being at the level of mini hospital, first-level or General hospital, they function at district level for the majority of emergency cases. Emergency health services (EHS) in Zambia are not linked to an existing pre-hospital care system (neither ambulance nor aero medical services). They are highly dependent on the in-hospital care of the Accident and Emergency (A&E) departments. Strengthening health systems in resource-limited countries has been a key goal of the World Health Organization (WHO) through the formulated health systems framework to set priorities in some or all of the six building blocks [19,20].

The WHO Hospital Emergency Assessment Tool (HEAT) instrument developed by the WHO in cooperation with the African Federation for Emergency Medicine (AFEM), a broad coalition from over 40 countries dedicated to securing high-quality emergency care for Africa, was used to conduct the assessments. This tool has been used in other LMICs to evaluate emergency care in facilities [21]. This tool was best suited for our study because it evaluates service delivery through key signal functions. Other tools such as the Emergency Care System Assessment, are for assessing the national rather than institutional level, while others focus on clinical aspects only, without infrastructure, equipment, and human resource components. The HEAT instrument allowed us to assess all aspects of the facilities in a holistic approach thereby providing areas for improvements. It was adopted by the WHO in 2017 to help improve overall health systems either at the single facility level or across multiple facilities at a regional or national level to gain a broader assessment of the entire emergency care system [4]. This study aimed to assess the capacity of district level hospitals to manage the increasing number of road traffic injury victims and to identify associated barriers to, and facilitators of, emergency trauma care in these facilities.

## Materials and methods

### Study setting

All six districts in Lusaka Province were included: Lusaka, Kafue, Chongwe, Luangwa,

Rufunsa, and Chilanga. A total of ten facilities were selected: all five level-one hospitals in Lusaka,

Nakachenje Mini-Hospital, Kafue General Hospital, Chongwe District Hospital, Luangwa District Hospital, and St Luke’s Mission Hospital.

### Study design

**Fig 1:**
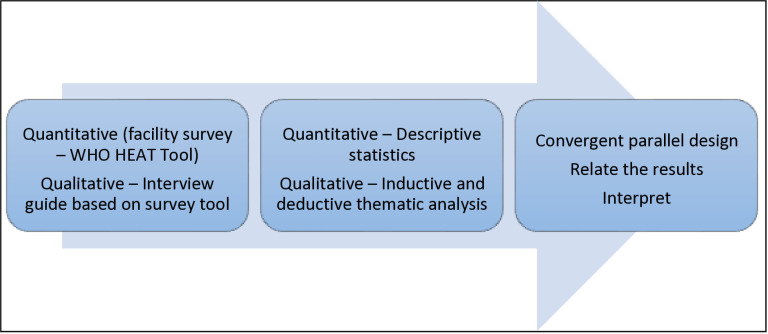
Methodology flow diagram.

A convergent mixed-method design was employed to assess the capacity of the district health facilities [22]. Quantitative and qualitative data were collected and analysed separately, but the results were compared and combined in the interpretation [23]. The quantitative and qualitative results were compared to identify key similarities and discrepancies as shown in “Fig 1”. Quantitative data were used to assess the facility’s capacity for emergency service provision to determine its readiness to manage optimally the rising number of road traffic victims. Qualitative interviews with emergency unit staff were conducted to identify key barriers to, and facilitators of, emergency trauma care.

### World Health Organization (WHO) hospital emergency assessment tool

#### (HEAT) Instrument.

The HEAT instrument is a standardized tool that assesses the capacity of health facilities to identify gaps that can be used to design interventions to strengthen emergency care delivery [24]. While the HEAT tool is primarily designed for assessing emergency units, it is not explicitly validated for research purposes. It was designed to evaluate the readiness and functionality of emergency units in delivering care during critical situations. Researchers have found it useful as a starting point for assessing emergency care systems.

The HEAT tool findings can be used to improve emergency care at specific facilities as well as the healthcare system. It has four domains: facility characteristics, human resources, clinical services, and signal functions. Signal functions measure the resource availability and skill set for performing emergency lifesaving interventions, which are rated on a three-point scale: adequate, some availability, and generally unavailable. The availability rating questions are used to assess resource and service capacities, specifically the ability to perform signal functions in the time frame needed for emergency care. It assesses both the demand-side and the supply-side factors for the service. For availability ratings scores of less than adequate (below 3), the factors that contribute to its deficiency can be further explored [23,24].

### Quantitative

#### Participant selection, recruitment, and data collection.

The participants were purposively selected and included; a hospital administrator, an information officer, a medical doctor, and the nurse in charge of the emergency unit. Consent was obtained and the HEAT instrument was used to collect data by the research team. Facility matrix information was also collected from the Health Management Information System (HMIS) I and II at each facility. A total of 33 interviews were conducted with a minimum of three hospital staff at each facility to obtain facility-level data. Data collection was done over five months from May to September 2023. Data were collected using Redcap, a secure web application for managing surveys and databases, designed specifically for clinical research studies and stored on a password-protected computer.

#### Data analysis.

The data were exported to Excel 2016 for cleaning and Stata/MP statistical software version 16.1 for descriptive analysis. Categorical variables were described using frequencies and proportions. Where appropriate, unless otherwise stated, a significance level of 0.05 was assumed. Excel tables were used to filter and determine the available key infrastructure, personnel and services. The availability of different categories of healthcare providers assigned to emergency units, including whether they rotated out of the unit or not, using frequencies and percentages were described. Frequencies were used to display the distribution of total annual emergencies and patient load for each facility. The distribution of inpatient beds, emergency beds and resuscitation beds in the study districts was also summarized. Additionally, statistics on the number of emergency operations performed annually were presented as the sum totals per district. These frequencies and percentages were generated using Stata software.

### Qualitative

#### Participant selection, recruitment, and data collection.

Interviews were conducted with a minimum of three healthcare providers at each facility, purposively sampled, each of whom had worked in the emergency unit for a minimum of one year. Written consent was obtained. Participants included medical doctors, medical licentiates, clinical officers, and nurses from the emergency department and high-dependency unit. The outpatient departments were substituted at the sites with no emergency unit. Interviews were conducted from June to September 2023. A total of 35 interviews were conducted face-to-face, 25 of which were done by PKM, while 10 interviews were performed by the research assistant after being trained and familiarized with the interview guide. A semi-structured interview guide developed using the WHO HEAT instrument for guidance. The interviews were carried out in English each one lasting between 30 and 45 minutes. Participants were encouraged to describe the barriers to, and facilitators of, emergency trauma care in their units, their experiences, thoughts, and actions. All interviews were audio-recorded.

#### Data analysis.

**Fig 2:**
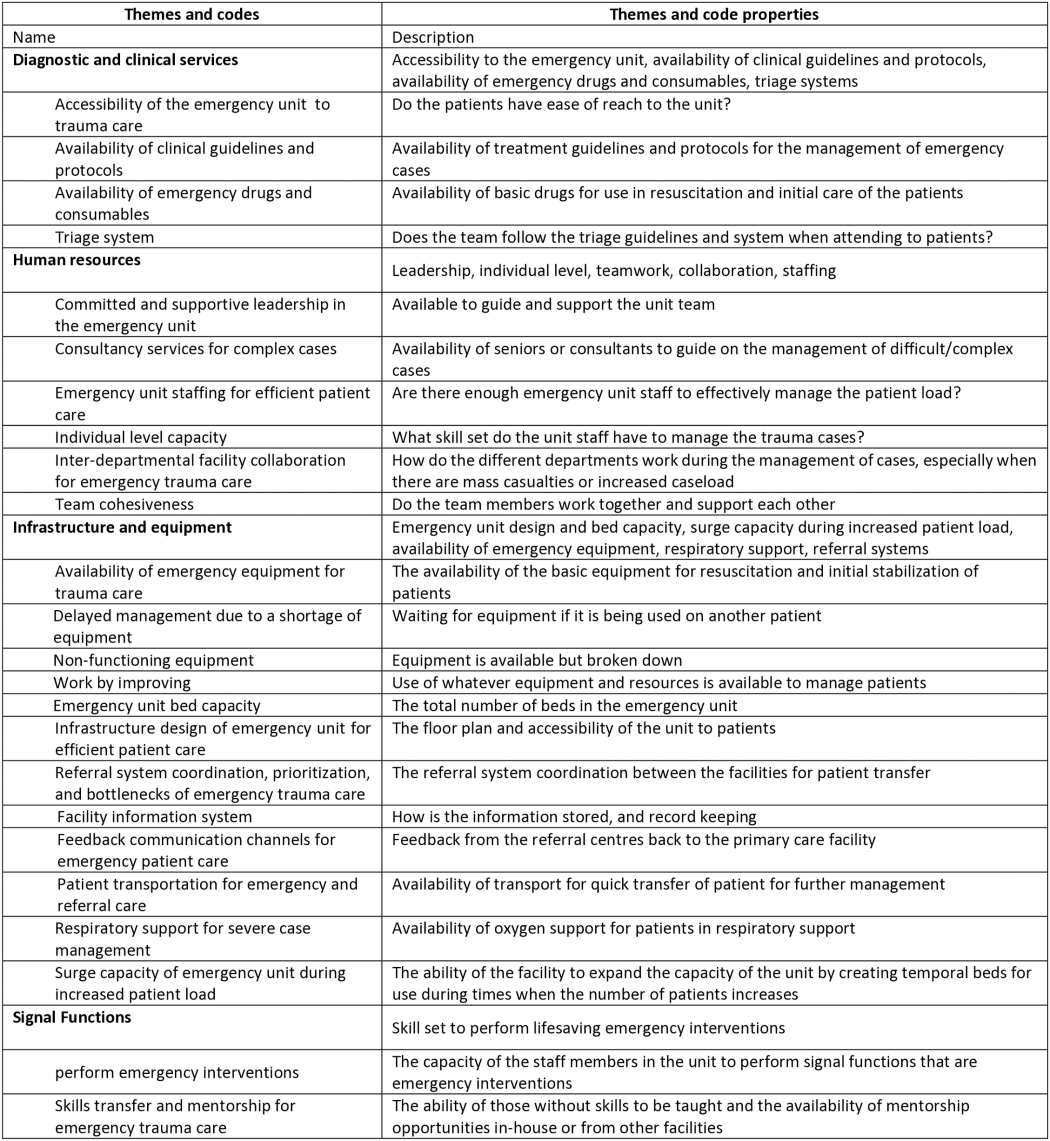
Codebook for barriers and facilitators to emergency trauma care at selected district hospitals in Lusaka, Province.

Interviews were transcribed by PKM and analysed using inductive and deductive thematic analysis – a method for identifying, analysing, and reporting themes within data [25]. The qualitative analysis software NVIVO (version 14, QRS International) was used to facilitate the data management, coding and analysis. This began by familiarization with the data and independently coding the first four transcripts and manually labelled portions of the text. TM and EM then read a selection of the transcripts for an independent analysis to familiarize themselves with the data, and afterward, discussions were held to review the initial codes and resolve any discrepancies with the coding and these were then applied to the whole material. A coding structure comprising thematic definitions and meaning was developed and imported into NVIVO 14 Pro for coding of the entire dataset “Fig 2”. Once the transcripts were coded, patterns and relationships guided the construction of themes based on the four predefined themes derived from the WHO HEAT tool as well as the subthemes that emerged inductively from the data “Table 1”. The initial codes were reviewed iteratively collapsing, realigning and clustering them into subthemes. The data analysis approach was done first deductively informed by the concepts in the WHO tool; and inductively in some of the main themes.

**Table 1:** Themes and subthemes for barriers to, and facilitators of, emergency trauma care.

Predefined themes (deductively derived)	Subthemes (inductively derived)
Infrastructure and equipment	Emergency unit design and capacity
	Surge capacity during increased patient load Availability of emergency equipment for trauma care Respiratory support for severe case management
	Referral systems
Human resources	Committed and supportive leadership
	Staffing and consultancy services Individual-level capacity and motivation Team cohesiveness Interdepartmental collaboration for emergency trauma care
Diagnostic and clinical services	Emergency unit accessibility
	Availability of clinical guidelines and protocols
	Emergency drugs and consumables
	Triage system
Signal functions	Performance of interventions
	Skills transfer and mentorship

### Ethical considerations

Ethical approval to conduct the study was given by the University of Zambia Biomedical

Research Ethics Committee (UNZABREC) (REF. No. 3637–2023) and the National Health Research Authority (NHRA) (REF No. NHRA000009/09/02/2023). Permission to conduct the study was granted by the Lusaka Provincial Health Director, the District Health Directors, and the Medical Superintendents for all the facilities. During data collection, participants were given relevant information about the study, and the benefits and risks were explained so that they made an informed decision about being involved. Written informed consent was obtained from all the participants at the beginning of each interview and separate permission was sought for recording the interviews. The interviews were carried out at the participant’s place of work. The confidentiality of the respondents was maintained throughout the study. All the participants were de-identified by assigning them participant codes using a unique identification number during the data collection and analysis process. Confidentiality and anonymity were assured in the reporting of statements.

### Inclusivity in global research

Additional information regarding the ethical, cultural, and scientific considerations specific to inclusivity in global research is included in the Supporting Information (S1 Checklist).

### Reflexivity

The main data collector was PKM with assistance from a trained research assistant, an orthopaedics and trauma surgeon trained in Zambia, and had no direct relationship with the participants. Her experience in the field of trauma care services meant she understood the challenges and requirements of delivering the services expressed by the study participants. However, we acknowledge that being a practicing surgeon in Zambia can introduce biases as regards the expected standards of service and care. To mitigate this, we chose to introduce a second data collector, who is not a medical professional, and we compared any observations of current practice to other LMICs as well as global standards for emergency trauma care rather than expected standards from any one professional. The interview transcripts were first analysed by PKM, who discussed the themes with TM and EM, with independent input on the findings from MM and JMZ.

## Results

### Demographic characteristics of the HEAT instrument participants

A total of 33 administrative and clinical members of staff across the six districts in Lusaka Province participated in the facility assessment. The primary role of the participants was as shown in “Table 2”.

**Table 2:** Characteristics of the HEAT tool participants from the facilities in terms of their primary role (n = 33).

Characteristics of participants	n (%)
**Number of participants**	33
**Primary role of the participant at the facility**	
Administrator	5 (15.1)
Information officer	9 (27.3)
Medical officer in charge of the unit	9 (27.3)
Sister in charge	10 (30.3)

### Demographic characteristics of the participants for qualitative interviews

Interviews were conducted with 35 participants from the ten facilities sampled, with an equal gender distribution. The characteristics of the participants are summarized in “Table 3.” The majority of the participants were registered nurses (n = 18, 51.3%) and all the participants and minimum of one to two years of experience dealing with trauma, emergencies, and more.

**Table 3:** Characteristics of the participants in terms of primary role, duration of working in the emergency unit and years of professional service (n = 35).

Characteristics	N=35 n (%)
**Number of participants**	35
**Primary role of the Participants**	
Specialist (General Surgeon)	1 (2.9)
Medical Doctor (Senior Resident)	10 (28.6)
Medical Licentiate	1 (2.9))
Clinical Officer General	4 (11.4)
Specialized Nurse (Trauma)	1 (2.9)
Registered Nurse	18 (51.3)
**Years worked in the Emergency unit**	
1–2	20 (57.2%)
3–5	13 (37.1%)
>5	2 (5.7%)
**Years in service**	
1–4	15 (42.9%)
5–10	13 (37.1%)
>10	7 (20.0%)

### Facility characteristics and matrices

The total population served by the facilities sampled was 2,206,301. The average distance to the nearest tertiary facility for Lusaka Province was found to be 10.8 kilometers. The average number of outpatient visits was 59,363 for adult patients and 3,378 emergency visits for children aged under five years. Two facilities had no designated emergency unit and only one had a high-dependency ward. The emergency unit for the majority of the facilities had 24-hour coverage supported by a pharmacy, laboratory, radiology, and theatre. The mean bed capacity was 4.7 and that of resuscitation beds was 2.6 for the province “Table 4”.

**Table 4:** Facility characteristics and matrices of the selected district facilities in Lusaka Province for the year 2022.

FACILITY CHARACTERICS	MEAN	TOTAL (N=10)	CHILANGA (n=1)	CHONGWE (n=1)	KAFUE (n=1)	LUANGWA (n=1)	LUSAKA (n=5)	RUFUNSA (n=1)
Size of population services by health facility(s)	265071.1	26507111	6205	229230	189948	38345	2153446	33537
Hours with emergency staff physically present in the unit	18.8	188	12	12	24	8	24	12
Total number of emergency visits including trauma patients (excluding Obstetrics)	59363.2	593632	12194	19531	30794	2844	516103	12166
Number of inpatient admissions	3106.4	31064	5678	1748	5933	605	3106	1570
Number of emergency operations	952.4	9524	753	1325	1027	1350	4981	88
Number of beds dedicated to emergency care (except beds for inpatients)	4.7	47	1	4	6	3	6	3
Number of beds dedicated to resuscitation of patients	2.6	26	1	2	1	2	3.6	2
Number of beds dedicated to inpatients use	109	1090	24	105	150	127	569	115

### Infrastructure and equipment

The facility assessment revealed that 40% had inaccessible unit locations limiting the possibility of emergency drop-offs by ambulances or private vehicles “Table 5”.

**Table 5:** Infrastructure and equipment availability and patient transport services at the selected facilities.

District Facility Assessment	Adequate n (%)	Some Availability n (%)	Generally Unavailable n (%)
**Infrastructure**			
Easy access to A & E	6 (60)	2 (20)	2 (20)
Waiting area	2 (20)	7 (70)	1 (10)
Triage area	1 (10)	6 (60)	3 (30)
Resuscitation area	4 (40)	4 (40)	2 (20)
**Equipment**			
Oxygen in the unit	7 (70)	1 (10)	2 (20)
Electro cardiac monitoring	1 (10)	3 (30)	6 (60)
Portable ultrasound	2 (20)	1 (10)	7 (70)
CT scan	0	0	10 (100)
X-ray	9 (90)	0	1 (10)
**Patient transport services**	0	7 (70)	3 (30)

#### Emergency unit design bed capacity.

In the interviews, the participants highlighted how the management of trauma victims is hindered by the lack of adequate dedicated beds for resuscitation and stabilization of acutely injured persons. This was supported by the majority of the facilities being rated as having some availability of a waiting area, triage area, or resuscitation area. This was compounded by a shortage of screening rooms and a lack of working areas to manage critically injured patients at the facilities.

“We have lack of space because we do not have a dedicated area” (IDI-08).

#### Surge capacity during increased patient load.

The ability to temporarily raise the capacity of emergency units during periods when patient load increased was reported as a facilitator. This helps to overcome the challenge by using extra mattresses or rooms during periods of increased patient load or mass casualties. However, this was reported as compromising the privacy and personal space of the patients because bedsiders and relatives of the victims crowded the space.

*“Sourcing for extra bed mattresses, sometimes we improvise and use floor mattresses, sometimes we improvise and put patients on one bed”* (IDI-03).

#### Emergency unit accessibility.

The location of the emergency units was considered a barrier by most of the participants. The inaccessibility of the units for rapid and efficient emergency trauma care was reported to contribute to delays in instituting care for the injured.

*“We would prefer it if we had a better location so that patients have easier access to us when we are needed”* (IDI-28).

#### Triage system.

Our assessment found that 70% reported how having a triage area and system has improved the organization of their units. They emphasized how using triage systems has greatly contributed to the efficient and safe care of their patients.

*“You would have patients waiting in the queues and then these are emergency cases so the patients would collapse while waiting to be attended to”* (IDI-28).

However, others reported that they do not use any triage system and that most of their team members lack knowledge about it. They made a plea for training and sanitization about triage to improve their patient care.

*“We need to teach people about triage, how we are supposed to do it and what we can do at the hospital…. sensitization on triage should be done so that people can be aware that once there is an emergency we will first attend to that emergency”* (IDI-06).

#### Respiratory support for severe case management.

Our assessment found that the oxygen supply was adequate at 70% of the facilities. The remaining facilities had shortages due to inadequate, faulty, or non-functioning oxygen machines. None of the facilities had centrally piped oxygen but relied on oxygen cylinders. Only one facility reported having a ventilator. However, despite having a ventilator, the oxygen supply cannot support mechanical ventilation because the demand for oxygen for other patients is equally high. They stated that the lack of ventilator support was a barrier to optimal care and has led to an increased number of referrals.

*“We have got a vent but most of the time we have a challenge with oxygen. If you say that we intubate we put that patient under the vent, and within some minutes the oxygen will finish then what will happen to those patients now who are there?”* (IDI-18).

#### Referral system.

The referral system was well organized and coordinated despite there being challenges with availability of ambulances at most of the facilities. Each facility has a command post that coordinates and records all the referral cases.

*“They have what we call command post; this is where the ambulance is based and there is the nurse as well, ambulance nurse to coordinate”* (IDI-01).

Our assessment found some availability of ambulances at 70% of the facilities, while they were generally unavailable at 30%. This was reported as a major barrier to the transfer of patients for further management. Participants stated that they are forced to use utility vehicles to transfer patients for specialist care.

*“We don’t have ambulances other than the vehicles that are used for administrative purposes. So those vehicles that are for administrative purposes are the ones that we are using as ambulances now”* (IDI-07).

Despite having ambulances, they stated that there are competing needs for trauma cases and obstetric emergencies, and obstetric cases take priority. Participants report that some ambulances are shared among several sub districts.

*“With these districts, you might find that maybe you are using one ambulance and priority most of the times is a pregnant woman so the ambulance maybe carries someone who is pregnant, the trauma patients remain”* (IDI-14).

### Human resources

An emergency core team was present in 40% of the facilities with the rest having rotating staff members supported by those from other departments. Specialist services were found to be lacking at most of the facilities, with general surgery being the only specialization that was adequate at 60% of them while orthopaedic surgeons were generally unavailable. Lusaka district had the highest healthcare provider-to-patient ratio despite recording the largest number of nurses, middle-level providers, and doctors. Most facilities had an average of two medical doctors assigned to the unit, with the majority being at senior resident level; the general hospital had only two doctors in the emergency unit who were also working in theatre “Table 6”.

**Table 6:** Human Resources: Emergency care clinical providers available in the emergency unit at each facility. The table shows the number of providers for each category of medical professional assigned to the emergency unit and the available consultation services at the facility for the year 2022.

Providers assigned to the emergency unit						
Provider type	District	*Non-Rotating		*Rotating		Total
		*Freq.*	*(Row %)*	*Freq.*	*(Row%)*	
***Total number of nurses/midwives***	Chilanga	15	(83.3)	3	(16.7)	18
	Chongwe	10	(62.5)	6	(37.5)	16
	Kafue	15	(65.2)	8	(34.8)	23
	Luangwa	10	(71.4)	4	(28.6)	14
	Lusaka	80	(67.2)	39	(32.8)	119
	Rufunsa	12	(50.0)	12	(50.0)	24
***Mid-level providers, clinical officers or advanced practice*** ***Nurses***	Chilanga	5	(50.0)	5	(50.0)	10
	Chongwe	8	(61.5)	5	(38.5)	13
	Kafue	5	(55.6)	4	(44.4)	9
	Luangwa	5	(55.6)	4	(44.4)	9
	Lusaka	50	(65.8)	26	(34.2)	76
	Rufunsa	6	(50.0)	6	(50.0)	12
***Medical doctors (without specialist training)***	Chilanga	1	(50.0)	1	(50.0)	2
	Chongwe	5	(55.6)	4	(44.4)	9
	Kafue	2	(50.0)	2	(50.0)	4
	Luangwa	3	(50.0)	3	(50.0)	6
	Lusaka	26	(52.0)	24	(48.0)	50
	Rufunsa	3	(60.0)	2	(40.0)	5
***Medical doctors with training in acute burn stabilization***	Chilanga	1	(50.0)	1	(50.0)	2
	Chongwe	5	(55.6)	4	(44.4)	9
	Kafue	0	–	0	–	0
	Luangwa	3	(50.0)	3	(50.0)	6
	Lusaka	25	(33.8)	49	(66.2)	74
	Rufunsa	2	(50.0)	2	(50.0)	4
***Fixed core emergency team***	Yes n (%)	No n (%)				
	4 (40)	6 (60)				
**Consulting Services**	**Adequate** n (%)	**Some Available** n (%)		**Unavailable** n (%)		
*General surgery*	6 (60)	1 (10)		3 (30)		
*Orthopaedics*	0	1 (10)		9 (90)		
*Anaesthetist*	4 (40)	5 (50)		1 (10)		

*Non-rotating staff: emergency unit staff permanently stationed in the unit

*Rotating staff: emergency unit staff from other departments who are assigned to the unit to augment the low staffing levels

#### Interdepartmental collaboration for emergency trauma care.

This was noted as a key facilitator through collaboration with other departments to beef up the staffing levels; nevertheless, the participants felt that having a permanent team would greatly improve their efficiency and effectiveness.

*“The whole team is involved and if we have a large number of victims even members of staff from other departments they also get involved to come and help us out”* (IDI-20).

#### Staffing and consultancy services.

Participants described how the low staffing levels negatively impact the management of patients due to increasing case load which makes them feel overburdened. The situation was said to be worse at night when only a skeleton staff was available to handle all the emergencies in addition to the inpatients.

*“It’s just one nurse against all those emergencies and one clinician and if there is something which is beyond them…. they only have two people on call in the night shift and they attend to all the departments at night”* (IDI-12).

#### Committed and supportive leadership.

Good governance was evident at most of the facilities with participants loving their working environment with management support. This has fostered team motivation for improved access to emergency care. Management initiatives such as creation of high-dependency wards for all emergency cases, with permanent staff providing 24 hours of critical care was a facilitator.

*“Ever since we created this HDU, there’s order in the way we are managing the patients because then there’s urgency in how we are managing them cause immediately they come, everyone is on their toes and making sure that things are done”* (IDI-17).

#### Team cohesiveness.

Participants described how the staff in the emergency unit are all dedicated to the management of emergency cases regardless of their primary role. A consensus among staff members that it’s all hands on deck whenever they receive any trauma victim. Emergency management of patients is well coordinated, and each member of the team clearly understands their role and is dedicated to the success of the team.

*“We normally have everyone on board starting with the doctors, the nurses, the radiologists; we have people from the anaesthetists who will come in also so we need to have the drivers also on standby”* (IDI-09).

#### Individual capacity and motivation.

The participants reported various challenges and strengths relating to their individual capacity in the management of emergency patients. Some of them explained how they rely on each other’s collective skills to manage emergencies due to the lack of mentorship programmes at their facilities. This approach fills in the individual skill gaps by using combined knowledge available within the team. They highlighted how patient management at some facilities is guided by peers rather than deliberate training of the staff in the emergency unit. Their lack of training sometimes affects the approach to case management, but despite their limited skill set, they are able to stabilize the patients and refer to further management if the need arises.

*“We are few on duty but we are putting together the knowledge that we have; we actually manage to help out the patients”* (IDI-01).

### Diagnostic and clinical services

All the facilities had 24-hour services for their laboratories and point-of-care tests. A basic haemoglobin check was available for emergency care and cross-matching could be done urgently. Urine dipsticks and glucose were available but participants reported a lack of stock and reagents as reasons why these services were not adequate. Tests like cardiac markers, coagulation profiles, and arterial blood gases were generally unavailable at all the facilities. Electrolyte assessment was available at only 50% of the hospitals and blood culture at just 70%. Clinical guidelines and protocols were found to be absent at most of the facilities. A trauma care checklist was missing at all the hospitals sampled. Protocols for ABCDs, medical resuscitation, burns, and volume resuscitation were present at 50% of the facilities “Table 7”.

**Table 7:** Diagnostic, point-of-care test and clinical management protocol availability at the selected facilities.

Characteristic		Availability Outcome	
	Adequate n (%)	Some availability n (%)	Generally Unavailable n (%)
**Laboratory testing**			
Haemoglobin	10 (100)	0	0
Full blood count	8 (80)	2 (20)	0
Coagulation profile	0	0	10 (100)
Electrolytes	0	5 (50)	5 (50)
Cardiac markers	0	0	10 (100)
Arterial blood gas	0	0	10 (100)
Cross-matching for blood	9 (90)	1 (10)	0
Blood cultures	0	7 (70)	3 (30)
**Emergency unit point of care**			
Urine dipsticks	4 (40)	6 (60)	0
Glucose	3 (30)	7 (70)	0
**Availability of Clinical Management** **Protocols**		**Yes (n)**	**No (n)**
Initial approach to ABCDs		5	5
Trauma care checklist		0	10
Medical resuscitation checklist		6	4
Neonatal resuscitation		2	8
Volume resuscitation		7	3
Burn care checklist		5	5

#### Availability of clinical guidelines and protocols.

The lack of checklists was evident at most of the facilities; hence they have nothing to refer to when managing their patients. Participants stated that they have no charts or printed treatment guidelines to use during the management of these patients.

*“Unfortunately I haven’t seen any guidelines; we just manage according to what we know best”* (IDI-31).

Their management is usually not guided by any standard protocols or clinical guidelines but by the knowledge from their professional training. This has made their work unstructured. One participant explained that this led to a lack of consistency and predictability in their approach.

*“Having a laid-out outline on how to handle anything I think makes it easier for everybody else because even for the person that is not sure of what to do they can easily refer to that for our patients”* (IDI-08).

However, some facilities reported that only the triage protocol is displayed on the walls to ensure a systematic approach to patient care. This protocol makes their work more organized and efficient. Efforts were being made to have all the protocols printed and stuck on the walls; however, the process has been delayed due to a lack of resources.

#### Emergency drugs and consumables.

Participants expressed challenges in the management of trauma patients due to the lack of essential medicines and consumables. Among them is the lack of splints, bandages, and plaster of Paris (POP) and have to use what is available, which is not standard care. Commodities are very scarce despite having support services open 24 hours a

day.

*“We do not have the splints needed, and when we have a fracture or something we are just improvising, we are using cardboard or just chitenges to immobilize the pelvis…. we do have access to theatre yes, it is a 24-hour theatre but uh scarcity of commodities makes it rather difficult to take them into the theatre at our hospital”* (IDI-16).

However, other participants reported that with introduction of the National Health Insurance Management Authority (NHIMA), they have seen an improvement in the availability of drugs and consumables.

*“We’ve been trying to make sure through NHIMA we’ve been getting resources through NHIMA to just buy and provide all the sutures and all the necessary consumables”* (IDI-17).

### Signal functions

Our assessment found some availability of cervical spine stabilization and fasciotomy interventions at 80% of the facilities. Fracture immobilization was not adequate at 50% of the facilities. Antibiotic availability for initial treatment of open fractures was adequate at 90% of the facilities “Table 8”.

**Table 8:** Signal functions and trauma interventions summary. This presents the availability rating at the selected facilities.

Characteristic n (%)		Availability Outcome	
	Adequate n (%)	Some available n (%)	Generally Unavailable n (%)
*Cervical-spine immobilization*	1 (10)	8 (80)	1 (10)
*Fasciotomy/escharotomy*	1 (10)	8 (80)	1 (10)
*Opiate analgesia admin*	6 (60)	3 (30)	1 (10)
*Fracture immobilization*	0	5 (50)	5 (50)
*Closed reduction of fracture*	4 (40)	5 (50)	1 (10)
*Administering antibiotics for open fracture*	9 (90)	1 (10)	0
*Initial wound care*	0	10	0

#### Perform emergency interventions.

Emergency interventions for trauma patients were reported to be well executed for air, breathing, and circulation procedures. Their failure to provide specific trauma interventions was attributed to a lack of skills and specialists, as reported by one participant:

*“We have general doctors that are there who also still need consultations for quite a number of things…. the surgeon we don’t have so most of the cases we refer them”* (IDI-06).

Due to inadequate skills, participants sometimes refer patients for further care. However, other participants reported that they were able to stabilize patients and provide emergency care after undergoing Basic Emergency Care (BEC) training. The training equipped them with the knowledge and skills required for basic emergency interventions.

*“I think that has also helped us a little bit and the thing is half of us or more than half are now trained in the Basic Emergency Care”* (IDI-19).

#### Skills transfer and mentorship.

Exchange programmes with tertiary hospitals are helping to improve their skill set through mentorship in selected facilities. Their competency in trauma management has been positively impacted by their experience at the tertiary facilities.

*“Recently we’ve had some exchange programmes with UTH and this has warmed up their skills. So they are competent in attending to trauma”* (IDI-16).

However, the staff members who have not been through the programme felt that only individuals were benefiting and not the facility. One participant reported that the knowledge is not translated to the rest of the team members once the staff member returns from their attachment.

*“What I’ve seen is people don’t come back and share that information. Those who didn’t have the opportunity to go there, meaning they will still remain blank”* (IDI-09).

#### Availability of emergency equipment for trauma care.

Participants pointed out how a lack of equipment has led to ineffective emergency trauma care in most of the facilities sampled. The survey revealed a lack of resuscitation equipment and monitors to manage emergencies, and the few that are available are non-functional, hence they are forced to improvise. Furthermore, the equipment within the facilities has to be shared by all departments, so this leads to delays in patient care.

*“We actually work by improvising…. So if that machine is being used by another patient means we are stuck, we need to wait for that patient to finish or the other people that are using it to finish on the patient”* (IDI-02).

## Discussion

This study is the first to comprehensively assess the capacity of emergency trauma care and capture the barriers and facilitators at district hospitals and their equivalent in Lusaka Province in Zambia. Our main quantitative findings showed that up to 40% of facilities had challenges in their units and inadequate working areas. Only two-thirds reported a functional triage system and access to adequate ambulance services. The qualitative findings highlighted a general challenge with a lack of trained personnel which was often resolved through collaboration between departments and personal motivation from staff with management support. Exchange and specific training programmes were highlighted as being valuable, however, the trainees need to transfer their knowledge to their colleagues in the respective facilities.

There was a significant difference across the six districts in terms of the capacity and preparedness of the emergency units, with some districts scoring higher than others in all the domains. The absence of a core trauma team was evident at the majority of the facilities with no permanent lead person in the emergency unit; hence, the emergency trauma system had no accountability or ownership.

### Facilitators of emergency care for road traffic injuries

Majority of the emergency units provided around-the-clock trauma care services with a well-coordinated organizational structure and designated triage and resuscitation areas. Twenty-four-hour accessibility of emergency services is paramount to any health system to ensure equitable coverage of trauma care with scarcely available specialized treatment. An organized and well-planned triage system is critical for efficient emergency diagnostic and clinical service delivery [26]. The emergency unit staff in our study explained how the introduction of triage protocols has led to an efficient and organized management system through prioritizing and quickly identifying disease conditions. There was a desire to improve the system-driven care at most of the facilities through a deliberate policy is required to ensure that equitable patient care is realized. A scoping review of trauma systems found that adopting a systems approach or organized management system through the introduction of a comprehensive system is associated with reduced mortality and morbidity related to trauma globally [27]. The improvement of care should be targeted at delivering meaningful, high-quality services that vulnerable populations can access during an emergency.

We found that at four facilities, a fixed core team was available ensuring that their trauma care services were well outlined, and the team leader was tasked with permanently overseeing the day-to-day coordination of the unit. This is supported by studies conducted in Australia which highlighted that the availability of a fixed core emergency or trauma team to manage and coordinate care ensures continuity and a smooth triage system flow [12]. When complex cases arise, fewer hiccups and difficulties will occur compared to having rotating junior medical officers or nurses unfamiliar with the trauma care system [10]. Like other LMICs, fragmented teams affect the organization and quality of care provided because they aim to just execute their shift-to-shift tasks with no accountability for the unit [28,29]. Consultants take on extra administrative roles leading to their inability to fully support the unit for complex cases as a result of task shifting. This can be mitigated by training junior staff members with the necessary skills – a strategy that has been noted in LIMCs [30]. The model of a fixed core team facilitated continuity and familiarity with patient care without competing demands from other tasks at the facilities.

High self-motivation and team dedication in providing timely emergency care by prioritizing the available resources was evident despite the high workload, the unit staff were resilient and quickly adapted to cope with mass casualties or increased patient attendance. Resilience is important in healthcare as shown by a study in Norway, where the healthcare system adapts to challenges and changes to ensure that quality of care is maintained through individual and system adaptive capacity [31]. Full management support at the facilities translated into a good working environment despite the many daily challenges faced by the teams. Studies have shown that leadership training, when incorporated into the trauma courses for healthcare providers, leads to improvement in the service delivery of emergency units and with notably patient-improved outcomes. Well-organized leadership improves the trauma care processes, and the highly motivated and dedicated team members facilitate timely management of the increased patient load [32].

To guarantee access to emergency care in Africa, the governance mechanisms require the establishment of legislation that should be embedded in the law for equitable care [33]. A multicountry study in Africa highlighted how despite gaps in governance, improvement through policy development can ensure prioritization of access to injuries and trauma care. Local governance initiatives through resource mobilization at some facilities led to the creation of a high-dependency unit (HDU), which has greatly improved their team efficiency and patient care. Participants highlighted how these initiatives are being used to ensure comprehensive treatment of the injuries with a dedicated team that is available throughout the 24-hour care within Lusaka Province. Furthermore, when the need for specialist care arises, the facilities are well equipped for transferring to the nearest higher-level care within the shortest time with support from auxiliary staff. Periodic assessment of health systems governance structures for trauma care in LMICs helps to document gaps for improvement through the introduction of trauma leadership courses for emergency staff [14].

Team cohesiveness was pivotal in their day-to-day service delivery, as seen in their coordinated management and dedication to the success of the unit. Studies performed on effective inter-professional leadership training programmes in trauma resuscitation, whether formal or informal, are valuable for emergency units in achieving proficiency. Local research has been encouraged in LMICs for researchers who understand their context and can design interventions to suit their emergency care systems [34]. The role of a team leader was well executed at one of the district facilities, which was reported as being the champion for trauma response. Continuous quality improvement (CQI) initiatives are being used worldwide to strengthen the healthcare system and a model of using a champion was conducted in Haiti at three facilities. A qualitative study among healthcare workers involved in CQI found that implementation was facilitated by the presence of a “champion”, who involved the facility staff in designing and effecting change to improve the system of care in an organization [35]. This model could be used in our setting to improve emergency trauma care systems by identifying champions with attributes of being passionate, committed, and results oriented. Borrowing from other health-strengthening strategies for improved care, the use of champions has been employed to advocate team efficiency and coordination in the management of patients in the emergency units where senior support is a challenge [36]. The champion ensures the prioritization of available resources in the face of competing demands and human resource challenges for optimal care, which has enjoyed success in community-based interventions [37].

### Barriers to emergency care for road traffic injuries

As the trauma burden continues to grow in LMICs, there is a critical disparity between the needs of the population and the human resources available, especially at the district level where the demands are high, similar to our findings [38]. The human resources domain established that qualified specialist doctors such as surgeons, anaesthetists, and obstetricians were either not available or lacking to institute emergency interventions compared to the number of resident medical officers providing care. Some facilities have at least one surgeon and an obstetrician, but availability varied across the districts. This is not unique to Zambia, as has been reported at district facilities in Cameroun, which has equally recorded an increase in the surgical burden of trauma and injuries with no specialist support [39]. Furthermore, Tanzania highlights how a shortage of triage personnel leads to work overload and fatigue resulting in reduced quality of healthcare services. The availability of trained personnel is critical to emergency care, and training lower-level health workers has greatly improved signal function performance [19,29].

**Fig 3:**
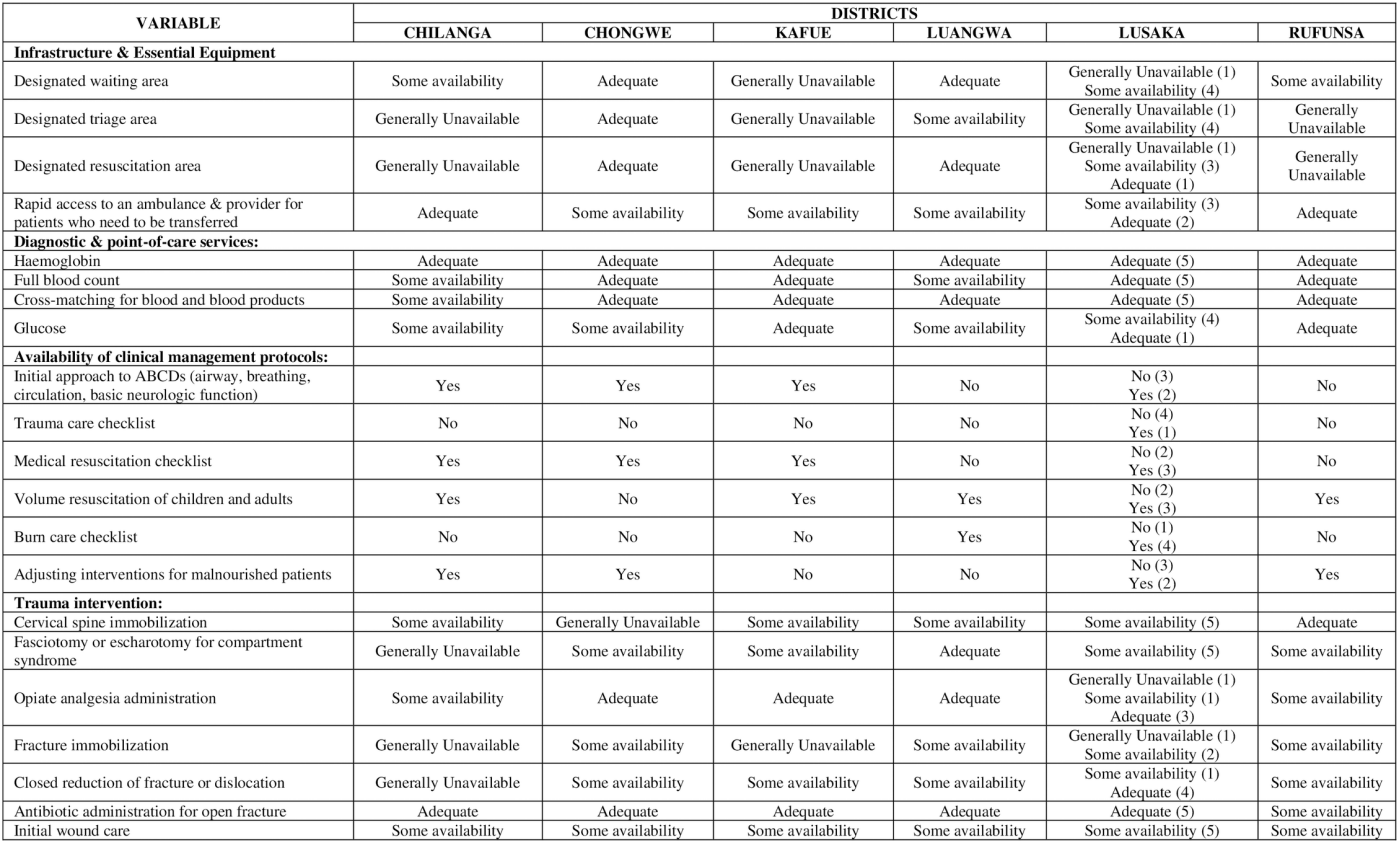
Comparative facility level availability of infrastructure, equipment, diagnostic and point of care tests, case management protocols and trauma interventions across the selected Hospitals in Lusaka, Province.

A critical lack of essential point-of-care test kits, emergency drugs, and trauma equipment across all the facilities “Fig 3”. This is not unique to our study, as seen from emergency capacity assessments done for LMICs in sub-Saharan Africa and Asia, which have revealed a scarcity of essential and critical emergency trauma equipment such as cervical collars, and fracture immobilization and stabilization splints, which compromises the standard of emergency care [40]. The unavailability of drugs and consumables posed a great challenge in the management of trauma patients and the emergency units relied on supplies from other departments to cushion their lack. Participants further explained how they had resorted to improvising for initial fracture stabilization by using cardboard boxes or cloth to provide emergency trauma care services. However, the introduction of the National Health Insurance Management Authority (NHIMA) in 2019, for universal health coverage (UHC) in Zambia has been a game changer, with increased access to drugs in pharmacies, advanced testing, and thus improved diagnoses at the health facilities.

There was a lack of basic ultrasound services at 70% of the facilities, leading to delays in diagnosing intra-abdominal trauma, or an inability to do so, with the potential risk of inadequate management and poor outcomes for the patients. In Zimbabwe studies to explore barriers to the provision of radiological services equally found that poor basic radiology equipment and infrastructure hampers patient safety and the quality of service delivery [41]. All facilities in our study had some availability of X-ray services but no CT scan machines. This mismatch of human resources and availability of equipment prevented the optimal provision of specific trauma care services, as seen in other LMICs like India [33].

Healthcare provider skill sets were found to be a barrier to optimal care, with the majority emphasizing that they had never undergone any formal training for emergency care. A review of existing trauma capacity conducted by the College of Surgeons of East, Central, and Southern Africa (COSECSA) also found that strengthening trauma-specific components of emergency care requires training in surgery, orthopedics, and neurosurgery to transform outcomes of accident victims in low-resource settings where the majority of the district hospitals lack specialized care [39–41]. Primary trauma care (PTC) courses for emergency unit teams in LMICs have resulted in enhanced service delivery, skills development, and overall reorganization of care for the acutely ill and injured during the initial care [42]. Human resource training in trauma care is critical to address the increasing burden and should therefore be integrated into the national health agenda [43].

The implementation of initiatives such as the WHO BEC (Basic Emergency Care) course at selected facilities had a positive impact, with participants calling for a rollout to all facilities with the help of government support [42,44]. These findings provide an understanding of how health system strengthening is being done for trauma care through mentorship programmes such as BEC, in conjunction with tertiary hospitals, to ensure skills transfer for improved services at the district level. Furthermore, low-cost training, which includes all departments, helps to improve knowledge and skills in trauma care and resuscitation, which has been done in other LMICs such as Tanzania, Ghana, Kenya, and India [9].

Significant variations of signal functions were found at the facilities depending on the on-duty staff, with a reduced number during the night and at weekends. The current imbalances in trained skilled health professionals and available resources found at the majority of the facilities, can be seen from other LMICs such as India where an urgent resolution has been made to strengthen trauma care facilities by addressing these gaps [45]. With the great need for specialists at the district level, the current establishment do not have these positions, therefore it is difficult to deploy the necessary cadres there unless they are in administrative positions. Signal functions at the majority of the facilities showed some availability for trauma interventions. Basic initial resuscitation procedures were well executed, but specific interventions such as fracture immobilization, fasciotomy, and closed fracture reduction were limited in terms of skill set. The availability of treatment guidelines promotes minimum standards for emergency care in a comprehensive and replicable manner worldwide [46]. Participants made a plea for all hospitals to be supplied with guidelines and protocols through support from the Ministry of Health. Deliberate government policies on improving emergency care are needed, with budgetary support integrated as part of the standardized training for all first-line providers, tailored to the needs and skills required for improved emergency care [47,48].

## Conclusion

Our findings indicate that facilities in Lusaka Province have a moderate capacity for emergency trauma care, although considerable variability exists among them. Inadequate emergency care space, medical supplies, and technologies, as well as skilled human resources, were the major barriers to emergency trauma care at the facilities, while good leadership, governance, and dedicated medical staff were the key facilitating factors. To manage the rising number of emergency trauma cases in Lusaka, there is a need to increase the proportion of facilities with adequate emergency unit infrastructure, bed capacity, triage system, and ambulance availability. In terms of human resource availability, the limited skilled human resources require training in trauma care skills as a priority. A collaborative effort is required on the part of health workers and the government to strengthen emergency trauma services at the district level and ensure a holistic, sustainable change in capacity building and budgetary support. A deliberate policy shift to introduce the use of core trauma teams to champion trauma care through basic training as an intervention in all primary health facilities is of utmost importance.

### Strengths and limitations of this study

We implemented an exhaustive sampling approach for the selection of facilities, whereby all district level hospitals within Lusaka Province providing in-hospital trauma care were surveyed, allowing for a comprehensive evaluation. Integrating the findings allowed an in-depth understanding of the complexity of the emergency trauma care health system through different lenses, highlighting the focus on improving care using the WHO HEAT instrument. Our use of deductive and inductive analyses enabled us to contribute new knowledge through subthemes that emerged from our data. The information collected was comprehensive due to the diversity of the range of healthcare workers included, which ensured a holistic view of the healthcare system from different perspectives [49]. To reduce the burden of data collection on the participant’s, questions on clinical data for each section were only asked to the specific unit head.

The subjective nature of our assessment presents the potential for response bias. We sought the information from the most appropriate staff members from hospitals, which sometimes leads to potential over- or under-reporting [50]. Access to statistical records for emergency patients was difficult because the current information systems do not separate them from all outpatient cases seen. To mitigate this, specific heads of clinical areas were interviewed independently. Furthermore, regarding our data collection methods, we note that observations only took place over one or two days, and it would be ideal to observe over a longer period covering different days of the week, given that activities and functions may vary across a given time. Since these observations served to validate contextual information given by the interviewees, however, this was partly mitigated by using a mixed-methods design to triangulate the data and gain new insights [51].

## Supporting information

S1 Checklist Inclusivity in global research. (PDF)

S1 Data Quantitative dataset for all the information collected and analysed for the study. (XLSX)

S2 Data Qualitative data and analysis for the interviews conducted in this study. (NVP)
