# Supplementary material for: Are we ready? Emergency unit capacity at selected district level hospitals in Lusaka Province, Zambia: Barriers and facilitators for improving trauma care: a mixed methods approach
Source: PLOS Glob Public Health. 2025 May 9;5(5):e0004382. doi: 10.1371/journal.pgph.0004382 (PMC12064030; doi:10.1371/journal.pgph.0004382)
Supplement: S1 Checklist — (PDF) [file pgph.0004382.s001.pdf]

Inclusivity in global research

PLOS' policy on inclusivity in global research aims to improve transparency in the reporting of research performed outside of researchers' own country or community and ensures that PLOS publications reporting global research adhere to high standards for research ethics and authorship. Authors of relevant research articles may be asked to complete the questionnaire below, which outlines ethical, cultural, and scientific considerations specific to inclusivity in global research. This questionnaire may be requested when researchers have travelled to a different country to conduct research, if research uses samples collected in another country, research with Indigenous populations or their lands, or if research is on cultural artefacts. Researchers travelling to another country solely to use laboratory equipment will not normally be required to complete the questionnaire. However, the questionnaire can be requested at the journal's discretion for any submission – if you have been requested to complete this questionnaire by the PLOS journal you submitted to, please do so.

Please complete the questionnaire below and include this as a Supporting Information file with your manuscript. Note that if your paper is accepted for publication, this checklist will be published with your article in the supporting information files. Please ensure that you reference the checklist in the main body of your manuscript. We suggest adding a subsection 'Inclusivity in global research' to your Methods section and adding the following sentence: "Additional information regarding the ethical, cultural, and scientific considerations specific to inclusivity in global research is included in the Supporting Information (SX Checklist)"

The questions have been designed to be applicable to a wide range of study types, and there are subsections for both human subjects research and non-human subjects research. If any of the questions are not relevant to your research please mark them as "N/A" as appropriate.

Ethical considerations, permits and authorship

This section is applicable to all research types.

Provide details as to who granted permissions and/or consent for the study to take place in the Methods section of your manuscript. This should include the names of **all** ethics boards, governmental organizations, community leaders or other bodies that provided approval for the study. If individuals provided approval refer to these people by their role or title but do not list their name(s).

Reported on page number 12 : Ethical approval to conduct the study was given by the University of Zambia Biomedical Research Ethics Committee (UNZABREC) (REF. No. 3637-2023) and the National Health Research Authority (NHRA) (REF No. NHRA000009/09/02/2023). Permission to conduct the study was granted by the Lusaka Provincial Health Director, the District Health Directors, and the Medical Superintendents for all the facilities. These have all be submitted as S6, S7 and S9 supporting information)

If there were any deviations from the study protocol after approval was obtained please provide details of these changes in the Methods section of your manuscript.

Reported on page number: None

Did this study involve local collaborators that are residents of the country where the research was conducted or members of the community studied? If you do not have any authors from said communities, please provide an explanation for this below.

The principal investigator and two other authors (the second and third) are all local residents of the country where the study was done. This is evidentin the authorship list on the title page.

Everyone listed as an author should meet PLOS' criteria for authorship and all individuals who meet these criteria should be included in the author byline, rather than the acknowledgements. For further information please see the journal's Authorship Policy.

Everyonr listed as author meets the PLOS' criteria for authorship.

Human subjects research (e.g. health research, medical research, cross-cultural psychology)

Did you obtain written informed consent from a representative of the local community or region before the research took place? How did you establish who speaks for the community? Details of written informed consent obtained from study participants should be reported separately in the Methods section of your manuscript.

Written informed consent and authorization was obtained from the Provincial Health Director, the local District Health Directors for each distrist and the Medical superintendants at each facilty (submitted as supporting information S8 appendix)

Informed written consent was obtained from each participant included in the research as outlined on page 12.

How did members of the local community provide input on the aims of the research investigation, its methodology, and its anticipated outcome(s)?

The local district health directors and heads of the facilities were briefed about the research aims and methodology prior to obtaining authorisation and were accorded an opportunity to give input or feedback. The were also consulted on what their views were about the research questions during the data collection and anticipated outcomes were discussed.

When engaging with the local community, how did you ensure that the informed consent documents and other materials could be understood by local stakeholders?

The informed consent documents were reviewed with the heads of the instistutions and the targeted population and explained in detail to emsure the materials were understood by all. Furthemore, all the participants were health professionals with tertiary level of education and conversant with the official language English. The consent forms were reviewed with each participant during the recruitment process

and they were given time to read through the documents and any querries or clarifications were addressed before written constent was obtained.

Will the findings of the research be made available in an understandable format to stakeholders in the community where the study was conducted (e.g. via a presentation, summary report, copies of publications, etc.)? Please provide details of how this will be achieved.

The finding of this research will be disserminated to all the facilities through a wriiten report but also a presentation to explain the findings and recommendations. Copies of the publication will be share with the provincial and district offices and preseation will be made at local conferences and symposiums. These findings will also be shared the surgical society of Zambia and Zambia orthopaedics and trauma associations at the annual conferences soon after publication of the manuscript.
The ethics board also requires that the publication be submitted to them for their records, archiving and dissermination of all approved studies.

Non-human subjects research using specimens/ animals collected as part of the study, or those housed in archival collections. Examples include archaeology, paleontology, botany and zoology.

Did the permission you obtained from a local authority to perform the study include an agreement on access to outputs and benefit sharing? This may include procedures to enable fair distribution of the benefits and resources arising from the research performed. Please include any details of Prior Informed Consent and Benefit Sharing Agreements obtained. These may be required by field-specific regulations, for example the Convention on Biological Diversity (CBD) and the associated Nagoya Protocol.

N/A

If the material used in your study was imported, please A) provide the year it was imported and B) indicate whether permits were obtained to import/export the materials used, C) provide details of any permits obtained. If this information is not available, please indicate this.

N/A

If you used archival specimens, please state how the material used in your study was acquired by the institute it is held in and provide details of any permits obtained for the original excavations/ sample collection. If this information is not available, please indicate this.

N/A

How was the potential cultural significance of the materials collected in your study to local communities considered in your research design? Were Indigenous peoples and/or local researchers and institutions involved with archaeological excavations / collection of specimens? If so, please provide a description of their involvement.

N/A

If your manuscript includes photographs of human remains please indicate whether authors obtained permission from descendants or affiliated cultural communities to do so.

N/A
